# MacaqueNet: Advancing comparative behavioural research through large‐scale collaboration

**DOI:** 10.1111/1365-2656.14223

**Published:** 2025-02-11

**Authors:** Delphine De Moor, Macaela Skelton, MacaqueNet, Federica Amici, Malgorzata E. Arlet, Krishna N. Balasubramaniam, Sébastien Ballesta, Andreas Berghänel, Carol M. Berman, Sofia K. Bernstein, Debottam Bhattacharjee, Eliza Bliss‐Moreau, Fany Brotcorne, Marina Butovskaya, Liz A. D. Campbell, Monica Carosi, Mayukh Chatterjee, Matthew A. Cooper, Veronica B. Cowl, Claudio De la O, Arianna De Marco, Amanda M. Dettmer, Ashni K. Dhawale, Joseph J. Erinjery, Cara L. Evans, Julia Fischer, Iván García‐Nisa, Gwennan Giraud, Roy Hammer, Malene F. Hansen, Anna Holzner, Stefano Kaburu, Martina Konečná, Honnavalli N. Kumara, Marine Larrivaz, Jean‐Baptiste Leca, Mathieu Legrand, Julia Lehmann, Jin‐Hua Li, Anne‐Sophie Lezé, Andrew MacIntosh, Bonaventura Majolo, Laëtitia Maréchal, Pascal R. Marty, Jorg J. M. Massen, Risma Illa Maulany, Brenda McCowan, Richard McFarland, Pierre Merieau, Hélène Meunier, Jérôme Micheletta, Partha S. Mishra, Shahrul A. M. Sah, Sandra Molesti, Kristen S. Morrow, Nadine Müller‐Klein, Putu Oka Ngakan, Elisabetta Palagi, Odile Petit, Lena S. Pflüger, Eugenia Polizzi di Sorrentino, Roopali Raghaven, Gaël Raimbault, Sunita Ram, Ulrich H. Reichard, Erin P. Riley, Alan V. Rincon, Nadine Ruppert, Baptiste Sadoughi, Kumar Santhosh, Gabriele Schino, Lori K. Sheeran, Joan B. Silk, Mewa Singh, Anindya Sinha, Sebastian Sosa, Mathieu S. Stribos, Cédric Sueur, Barbara Tiddi, Patrick J. Tkaczynski, Florian Trebouet, Anja Widdig, Jamie Whitehouse, Lauren J. Wooddell, Dong‐Po Xia, Lorenzo von Fersen, Christopher Young, Oliver Schülke, Julia Ostner, Christof Neumann, Julie Duboscq, Lauren J. N. Brent

**Affiliations:** ^1^ School of Psychology, Centre for Research in Animal Behaviour University of Exeter Exeter UK; ^2^ Research Group for Human Biology and Primate Cognition, Biology Institute University of Leipzig Leipzig Germany; ^3^ Department of Comparative Cultural Psychology Max Planck Institute for Evolutionary Anthropology Leipzig Germany; ^4^ Faculty of Biology, Institute of Human Biology and Evolution Adam Mickiewicz University Poznan Poland; ^5^ Faculty of Science & Engineering, School of Life Sciences Anglia Ruskin University Cambridge UK; ^6^ Department of Population Health & Reproduction, School of Veterinary Medicine University of California at Davis Davis California USA; ^7^ Laboratoire de Neurosciences Cognitives et Adaptatives Strasbourg France; ^8^ Centre de Primatologie de l'Université de Strasbourg Niederhausbergen France; ^9^ Domestication Lab, Department of Interdisciplinary Life Sciences, Konrad Lorenz Institute of Ethology University of Veterinary Medicine Vienna Vienna Austria; ^10^ Program in Evolution, Ecology and Behavior, Department of Anthropology University at Buffalo Buffalo New York USA; ^11^ Division of Natural Sciences, Engineering and Mathematics, Department of Biology University of St. Thomas Houston Texas USA; ^12^ Animal Behavior & Cognition, Department of Biology Utrecht University Utrecht The Netherlands; ^13^ Department of Psychology and the California National Primate Research Center University of California at Davis Davis California USA; ^14^ Primatology Research Group, Research Unit SPHERES The University of Liège Liège Belgium; ^15^ Institute of Ethnology and Anthropology Russian Academy of Sciences Moscow Russia; ^16^ Wildlife Conservation Research Unit (WildCRU) University of Oxford Oxford UK; ^17^ Department of Sciences Roma Tre University Rome Italy; ^18^ Conservation Science and Outreach North of England Zoological Society Cheshire UK; ^19^ Animal Behaviour and Cognition Programme, National Institute of Advanced Studies Indian Institute of Science Campus Bengaluru India; ^20^ Department of Psychology University of Tennessee Knoxville Knoxville Tennessee USA; ^21^ Science Department North of England Zoological Society Chester UK; ^22^ FES Zaragoza National Autonomous University of Mexico Mexico City Mexico; ^23^ National Institute of Psychiatry Ramón de la Fuente Muñiz Mexico City Mexico; ^24^ School of Psychology Mexico City Mexico; ^25^ Fondazione Ethoikos Convento dell'Osservanza Radicondoli Italy; ^26^ Yale Child Study Center Yale School of Medicine New Haven Connecticut USA; ^27^ Animal Behaviour and Cognition Programme National Institute of Advanced Studies Bengaluru India; ^28^ Department of Zoology Kannur University Kannur India; ^29^ Department of Anthropology, Durham Cultural Evolution Research Centre Durham University Durham UK; ^30^ Cognitive Ethology Lab German Primate Center Göttingen Germany; ^31^ Department for Primate Cognition Georg‐August‐University Göttingen Göttingen Germany; ^32^ Primate Cognition Göttingen Germany; ^33^ Department of Anthropology Durham University Durham UK; ^34^ Department of Behavioral and Cognitive Biology University of Vienna Vienna Austria; ^35^ Department of Anthropology Princeton University Princeton New Jersey USA; ^36^ The Long‐Tailed Macaque Project Sorø Denmark; ^37^ Behavioural Ecology Group, Department of Biology Univeristy of Copenhagen Copenhagen Denmark; ^38^ Oxford Wildlife Trade Research Trade Group Oxford Brookes University Oxford UK; ^39^ Behavioural Ecology Research Group, Institute of Biology University of Leipzig Leipzig Germany; ^40^ Department of Human Behavior, Ecology and Culture Max Planck Institute for Evolutionary Anthropology Leipzig Germany; ^41^ School of Biological Sciences Universiti Sains Malaysia Gelugor Malaysia; ^42^ School of Animal, Rural and Environmental Sciences Nottingham Trent University Southwell UK; ^43^ Department of Zoology, Faculty of Science University of South Bohemia České Budějovice Czech Republic; ^44^ Sálim Ali Centre for Ornithology and Natural History Coimbatore India; ^45^ Départment d'Anthropologie, Faculté des Arts et des Sciences Udem Montréal Quebec Canada; ^46^ Department of Psychology University of Lethbridge Lethbridge Alberta Canada; ^47^ Whitelands College Roehampton University London UK; ^48^ International Collaborative Research Center for Huangshan Biodiversity and Tibetan Macaque Behavioral Ecology, School of Resource and Environmental Engineering Anhui University Hefei China; ^49^ School of Resource and Environmental Engineering Anhui University Hefei China; ^50^ ZooParc de Beauval et Beauval Nature Saint‐Aignan France; ^51^ Wildlife Research Center Kyoto University Inuyama Japan; ^52^ School of Psychology University of Lincoln Lincoln UK; ^53^ Wildlife Park Goldau Goldau Switzerland; ^54^ Austrian Research Center for Primatology Ossiach Austria; ^55^ Forest Conservation Department Hasanuddin University Makassar Indonesia; ^56^ NTU Psychology Nottingham Trent University Nottingham UK; ^57^ Applied Behavioural Ecology and Ecosystems Research Unit University of South Africa Pretoria South Africa; ^58^ Department of Psychology, Centre for Comparative and Evolutionary Psychology University of Portsmouth Portsmouth UK; ^59^ Srishti Manipal Institute of Arts Design and Technology Bengaluru India; ^60^ CLLE, Université de Toulouse, CNRS Toulouse France; ^61^ Department of Anthropology University of Georgia Athens Georgia USA; ^62^ Institute for Evolutionary Ecology and Conservation Genomics Ulm University Ulm Germany; ^63^ Department of Biology University of Pisa Pisa Italy; ^64^ Laboratoire de Psychologie Sociale et Cognitive Centre National de la Recherche Scientifique et Université Clermont‐Auvergne Clermont‐Ferrand France; ^65^ Istituto di Scienze e Tecnologie della Cognizione Consiglio Nazionale delle Ricerche Rome Italy; ^66^ Foundation for Ecological Research Advocacy and Learning Morattandi Villupuram India; ^67^ Anthropology Program, School of Anthropology, Political Science and Sociology Southern Illinois University Carbondale Carbondale USA; ^68^ Department of Anthropology San Diego State University San Diego California USA; ^69^ Department of Life Sciences University of Roehampton London UK; ^70^ Primate Social Evolution Group, German Primate Center Leibniz Institute for Primate Research Göttingen Germany; ^71^ Behavioral Ecology Department University of Göttingen Göttingen Germany; ^72^ Anthropology Central Washington University Ellensburg Washington USA; ^73^ School of Human Evolution and Social Change and Institute of Human Origins Arizona State University Tempe Arizona USA; ^74^ Biopsychology Laboratory, Institution of Excellence University of Mysore Mysuru India; ^75^ Institut pluridisciplinaire Hubert Curien (UMR 7178), Centre national de la recherche scientifique Université de Strasbourg Strasbourg France; ^76^ Natural Resources Institute University of Greenwich Kent UK; ^77^ Research Centre for Evolutionary Anthropology & Palaeoecology, School of Biological and Environmental Sciences Liverpool John Moores University Liverpool UK; ^78^ Department of Anthropology Northern Arizona University Flagstaff Arizona USA; ^79^ Department of Primate Behavior and Evolution Max‐Planck Institute for Evolutionary Anthropology Leipzig Germany; ^80^ Department of Neurosurgery Emory University Atlanta Georgia USA; ^81^ School of Life Sciences Anhui University Hefei Anhui China; ^82^ Nuremberg Zoo Nürnberg Germany; ^83^ UMR7206 Eco‐Anthropology CNRS‐MNHN‐Université Paris Cité Paris France

**Keywords:** comparative research, data sharing, database, *Macaca*, primates, repository, social networks, team science

## Abstract

1. There is a vast and ever‐accumulating amount of behavioural data on individually recognised animals, an incredible resource to shed light on the ecological and evolutionary drivers of variation in animal behaviour. Yet, the full potential of such data lies in comparative research across taxa with distinct life histories and ecologies. Substantial challenges impede systematic comparisons, one of which is the lack of persistent, accessible and standardised databases.
2. Big‐team approaches to building standardised databases offer a solution to facilitating reliable cross‐species comparisons. By sharing both data and expertise among researchers, these approaches ensure that valuable data, which might otherwise go unused, become easier to discover, repurpose and synthesise. Additionally, such large‐scale collaborations promote a culture of sharing within the research community, incentivising researchers to contribute their data by ensuring their interests are considered through clear sharing guidelines. Active communication with the data contributors during the standardisation process also helps avoid misinterpretation of the data, ultimately improving the reliability of comparative databases.
3. Here, we introduce MacaqueNet, a global collaboration of over 100 researchers (https://macaquenet.github.io/) aimed at unlocking the wealth of cross‐species data for research on macaque social behaviour. The MacaqueNet database encompasses data from 1981 to the present on 61 populations across 14 species and is the first publicly searchable and standardised database on affiliative and agonistic animal social behaviour. We describe the establishment of MacaqueNet, from the steps we took to start a large‐scale collective, to the creation of a cross‐species collaborative database and the implementation of data entry and retrieval protocols.
4. We share MacaqueNet's component resources: an R package for data standardisation, website code, the relational database structure, a glossary and data sharing terms of use. With all these components openly accessible, MacaqueNet can act as a fully replicable template for future endeavours establishing large‐scale collaborative comparative databases.

## Graphical abstract

We present MacaqueNet, a global community of macaque researchers who developed the first publicly searchable, standardised database on affiliative and agonistic behaviour. This cross‐species database facilitates comparative research into the eco‐evolutionary drivers of animal behaviour. We describe its management and share its resources as a replicable template for other collaborative databases.

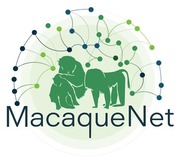

## 1 INTRODUCTION

Comparative studies are fundamental in understanding the biological basis of traits. They can reveal broad patterns in evolutionary and developmental history, past and current selective pressures and underlying mechanisms (Nunn, 2011; Tinbergen, 1963). Yet, a major limitation to comparative research is reaching a sufficiently large and diverse sample for reliable inference (Borries et al., 2016; Schneider et al., 2019). Comparative studies therefore typically rely on combining independent research on individual populations (Lukas & Clutton‐Brock, 2017).

There is a vast and ever‐accumulating amount of behavioural data on individually recognised animals (Sheldon et al., 2022). This is an incredible resource for comparative research, that can reveal fundamental principles on the ecological and evolutionary processes that shape animal behaviour. However, combining these rich behavioural data comes with substantial challenges (Pinter‐Wollman et al., 2013). The field of animal behaviour historically and currently still consists of many independent research groups collecting and managing data using similar yet slightly different methods (the ‘long tail’ of data: many small datasets that together represent the vast majority of existing data; Wallis et al., 2013). Choices regarding which behaviours to observe, how those behaviours are defined, and which methods are used to record them, result in a wide variation in behavioural data types and definitions. This complicates the creation of standardised databases on behaviour, compared with existing databases pooling relatively consistently defined and quantified data, such as life history and demography, morphological and ecological traits, population sizes and geographic ranges, social organisation and measures of biodiversity (SPI‐Birds: Culina et al., 2020; The Global Biodiversity Information Facility: Edwards et al., 2000; Co‐BreeD: Mocha et al., 2024; panTHERIA: Jones et al., 2009; COMADRE: Salguero‐Gomez et al., 2016; The Primate Life History Database: Strier et al., 2010; BIDDABA : Lebreton et al., 2010).

Compounding this challenge, most comparative studies in animal behaviour typically consist of one‐off comparative research projects, spearheaded by an individual research team and focused on a specific question. The generated databases are usually not conceived for general use, making them difficult to find or repurpose for future studies (O'Dea et al., 2021). For instance, data are often summarised into a single datapoint per species, according to the definitions and decisions of the research team and project. As such, the painstaking effort of searching the literature for suitable data, acquiring such data, and standardising the data and metadata across datasets needs to be repeated for each new comparative study (Poisot et al., 2019). A far more sustainable approach is to create enduring and reusable databases, with well‐defined protocols for (meta)data standardisation, archiving and accessing (Culina et al., 2020; Urbano & Cagnacci, 2021). Clear and concise guidelines for such databases are provided by the Findable, Accessible, Interoperable and Reusable (FAIR) guiding principles to ensure data are ‘FAIR’ (Wilkinson et al., 2016). The development of such databases has become a priority for many scientific fields and has led to the creation of big‐team science initiatives, bringing together scientists ‘across labs, institutions, disciplines, cultures, and continents’ (Forscher et al., 2022, p. 2). The aim of these collaborations is to enable large teams of researchers to collaborate more effectively and to accelerate scientific discoveries by making it easier to integrate and compare data from different sources.

Many of these team‐science endeavours follow the ‘Many Labs’ approach (Klein et al., 2014; e.g. ManyPrimates: ManyPrimates et al., 2019; ManyBabies: Visser et al., 2022), where research teams collaborate to each contribute data collected according to a standardised protocol to maximise comparability. This is a promising approach, and one that ideally should be adopted more broadly across long‐term research sites to allow for the identification of broad patterns across sites and study systems (Rubenstein & Abbot, 2017). Yet, it would be a waste not to use the 70+ years' worth of behavioural data that already exist (Purgar et al., 2022; Sheldon et al., 2022). Although complex, there is great potential in standardising existing data and making them usable for comparative research (Pinter‐Wollman et al., 2013).

Two big‐team initiatives on animal spatial data, Euromammals and Movebank, demonstrate the strength of collaborative efforts in standardising and centralising animal behaviour data. Since their inception in 2007 and 2008, respectively, these initiatives have grown into key repositories for thousands of animal movement datasets, supporting collaborative research and resulting in numerous publications (Kays et al., 2021; Kranstauber et al., 2011; Urbano & Cagnacci, 2021). More recently, similar initiatives have emerged to aggregate social behavioural data. The Animal Social Network Repository (ASNR) was the first comprehensive effort to consolidate social behaviour data across different animal species using FAIR principles (Sah et al., 2019). Since its publication, the ASNR database has been used for several comparative studies across a broad taxonomic range (e.g. Collier et al., 2022; Gagliardi et al., 2023), demonstrating the value of such databases for the scientific community. However, the ASNR database's diversity also presents challenges for comparative research as it contains unstandardised social data collected in various ways, with substantial variation in observation effort and capturing fundamentally distinct aspects of sociality. A second, recently developed database is DomArchive, which focuses on dominance interactions within groups of various taxa (Strauss et al., 2022). Social dominance is conceptually similar and measured fairly consistently across taxa (Strauss et al., 2022), and therefore lends itself well to comparative questions. However, a comprehensive understanding of social behaviour necessitates data on both affiliative and agonistic social interactions. Both types of interactions play crucial but distinct roles in shaping the dynamics and complexity of social systems. In contrast to dominance interactions, affiliative interactions are typically more varied in terms of both the types of behaviours and methods used to measure them, making it more complex to build a database of truly comparable data (De Moor et al., 2024; Ellis et al., 2019). One approach to overcoming this challenge is to collate data for taxa in which the observed behaviours and recording protocols are relatively uniform. These databases can then be integrated into an archive of databases, such as the EcoEvo data source catalogue (https://ckan‐ecoevo.d4science.org/, Culina et al., 2018), to ultimately create a comprehensive FAIR database of social behaviour.

Here, we introduce MacaqueNet (https://macaquenet.github.io/), a global grassroots collaboration that provides a platform for big‐team social behavioural research on macaque monkeys (*Macaca* spp.), and the MacaqueNet database, a cross‐species collaborative FAIR database, facilitating synthetic comparative research (Borries et al., 2016; Coles et al., 2022). To pave the way for future collaborative initiatives in comparative behavioural research, we describe how we initiated MacaqueNet, compiled and standardised data to create a relational database, implemented data sharing protocols and built a global community of collaborating researchers. With all core components openly accessible, MacaqueNet can act as a fully replicable template for other researchers interested in setting up collaborative cross‐species databases.

## 2 METHODS AND RESULTS

MacaqueNet connects researchers studying the social behaviour of macaques in diverse settings. The aim of MacaqueNet is twofold: to encourage and facilitate grassroots collaboration between independent research teams and to provide a lasting central platform for data archiving, standardising and accession. Here, we describe the establishment of MacaqueNet, from starting a large‐scale collective to the creation of a cross‐species collaborative database and the implementation of data entry and retrieval protocols. First, however, we illustrate the exceptional suitability of macaques as a taxon to initiate the construction of cross‐species databases of social behavioural data.

### 2.1 Why the genus *Macaca* lends itself to the creation of a comparative database of social behaviour

With 25 currently recognised extant species, *Macaca* is the most widely geographically distributed non‐human primate genus (Roos et al., 2019). Macaques are one of the most versatile and adaptable primates, exploiting very different environments, from the temperate, mountainous habitats of Morocco and Japan to the tropical forests of Southeast Asia (Thierry, 2007). Throughout this range, they live in areas of varying anthropogenic impact, from cities over natural habitats coming under increasing tourist and agricultural pressure, to pristine forests (Radhakrishna et al., 2012). This variation in habitats and climates is reflected in the species' ecology, mating system and social structure (the content, quality and patterning of social relationships between individuals belonging to the same social unit; Cords, 2012; Kappeler et al., 2019). Macaques are primarily frugivorous, range from being arboreal to semiterrestrial and encounter different levels of predation (Fleagle, 2013). Mating is polygynandrous and reproduction can be seasonal or year‐round, with substantial variation in male reproductive skew (biased distribution of offspring sired towards a few, usually high‐ranking, males) across species (Schülke & Ostner, 2008).

Despite this variation, macaques have a rather conserved social organisation (the size and composition of a social unit; Kappeler et al., 2019). They typically live in relatively large multi‐male multi‐female groups of approximately ten to a hundred individuals in the wild and up to several hundred in urban or provisioned populations (Cords, 2012). Females are philopatric and form the core of the group, clustered into multigenerational matrilines. Males typically disperse from their birth group around puberty to breed elsewhere and may even change groups several times in their life (De Moor et al., 2020). Both male and female macaques exhibit a wide range of social behaviours, many of which are shared across all species (Thierry, 2007). While social relationships tend to be kin biased and hierarchical to some extent, macaques show substantial variation in the patterns of affiliative and agonistic interactions. This remarkable diversity within unity makes macaques a compelling taxon for establishing a cross‐species database aimed at investigating the ecological and evolutionary processes underlying this variability (Balasubramaniam et al., 2018; Thierry, 2007).

Macaques are also excellent as a starting point to address the challenge of cross‐species behavioural databases. Many species of macaques have been extensively studied in the wild or naturalistic semi‐free ranging settings for years or even decades, with observed behaviours and recording protocols being relatively uniform across research sites, facilitating comparison (*Macaca assamensis*, Ostner & Schülke, 2018; *Macaca fascicularis*, Van Noordwijk & Van Schaik, 1985; *Macaca fuscata*, Nakagawa et al., 2010; *Macaca leonina*, Albert et al., 2013; *Macaca maura*, Okamoto et al., 2000; Riley et al., 2014; *Macaca mulatta*, Cooper et al., 2022; *Macaca nemestrina*, Ruppert et al., 2018; *Macaca nigra*, Duboscq & Micheletta, 2023; *Macaca radiata*, Sinha, 2005; *Macaca sinica*, Dittus, 1975; *Macaca sylvanus*, McFarland & Majolo, 2013; *Macaca thibetana*, Li et al., 2020). Japanese macaques (*Macaca fuscata*) were one of the first species for which researchers recognised and followed subjects individually, generating unprecedented levels of detail on their behaviour. The first published record on macaque behaviour dates back to 1956 (Kawamura, 1956). Since then, a body of research has leveraged macaque behavioural data, including comparative research across macaque species (Balasubramaniam et al., 2012, 2020; De Waal & Luttrell, 1989; Sueur et al., 2011; Thierry et al. 2000; Thierry, 2021), to greatly contribute to answering fundamental questions on the evolution, selective pressures and adaptive functions of social behaviour. For instance, macaque research has shed light on the evolutionary drivers and consequences of dominance hierarchies (Balasubramaniam et al., 2012, 2018; Bernstein & Sharpe, 1966; Neumann & Fischer, 2023; Simons et al., 2022; Van Noordwijk & Van Schaik, 1985), the influence of kinship on social structure (Berman & Thierry, 2010; Brent et al., 2017; De Moor et al., 2020; Schülke & Ostner, 2008; Widdig et al., 2016; Yamada, 1963), patterns of social ageing (Almeling et al., 2016; Sadoughi et al., 2024; Siracusa et al., 2022), the ultimate function of social relationships (Campbell et al. 2018; Ellis et al., 2019; McFarland & Majolo, 2013; Micheletta et al., 2012; Riley et al., 2014; Schülke et al., 2010; Singh et al., 2010; Young et al., 2014) and the impact of anthropogenic challenges on social structure (Balasubramaniam et al., 2020; Holzner et al., 2019; Kaburu et al., 2019; Morrow et al., 2019; Testard et al., 2021). Building on these strong foundations, MacaqueNet aims to further promote collaborative and comparative research on macaque social behaviour.

### 2.2 Creating MacaqueNet

MacaqueNet originated in 2017 as a small‐scale collaborative proposal for one specific study. From there, MacaqueNet developed and expanded to become a global grassroots network of macaque researchers. To mitigate the inherent bias towards researchers from Europe and the United States, and to facilitate the involvement of scientists from macaque‐range nations and historically underrepresented research communities we identified further potential collaborators through word of mouth and a literature search on Google Scholar. An initial email was sent to all first and last authors of papers that contained or pointed to the existence of social behavioural data on macaques, inviting them to contribute their data to a collaborative database. We asked the contacted researchers to forward the invitation to their relevant collaborators and to list those collaborators as co‐contributors of the data shared with MacaqueNet, ensuring everyone received proper credit. We also encouraged them to suggest other researchers who might be interested in joining the consortium. This inclusive approach allowed us to engage a broader community of researchers. As a result, the MacaqueNet community currently consists of 106 researchers based at 58 institutes across five continents. Beyond our data contributors, MacaqueNet is open to any interested researcher, regardless of whether they have contributed data. All members receive a quarterly newsletter, can join meetings, request data and propose new research directions.

### 2.3 The MacaqueNet relational database

**FIGURE 1:**
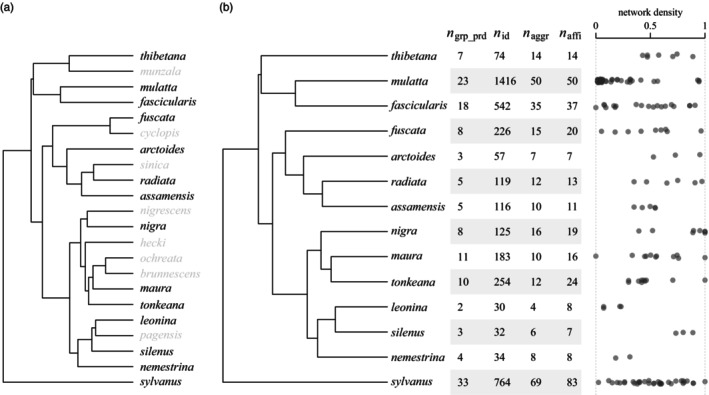
Summary of database content at the time of publication. (a) The MacaqueNet database currently contains data for 14 out of 25 recognised macaque species (note that the depicted phylogenetic tree (Arnold, Matthews & Nunn, 2010) does not include relatively newly described species: *Macaca selai*, *Macaca leucogenys* and *Macaca siberu*). (b) Overview of the number of group‐periods (i.e. a given study period for a given group), individuals and sociometric matrices for aggressive and affiliative behaviours for each species. As some individuals have been observed over multiple group‐periods, the number of individuals represents the number of unique individual datapoints but not necessarily the number of unique individuals. The dot plot on the right illustrates grooming network densities (the proportion of dyads that were observed grooming at least once), with each dot representing the density for one grooming sociometric matrix.

**FIGURE 2:**
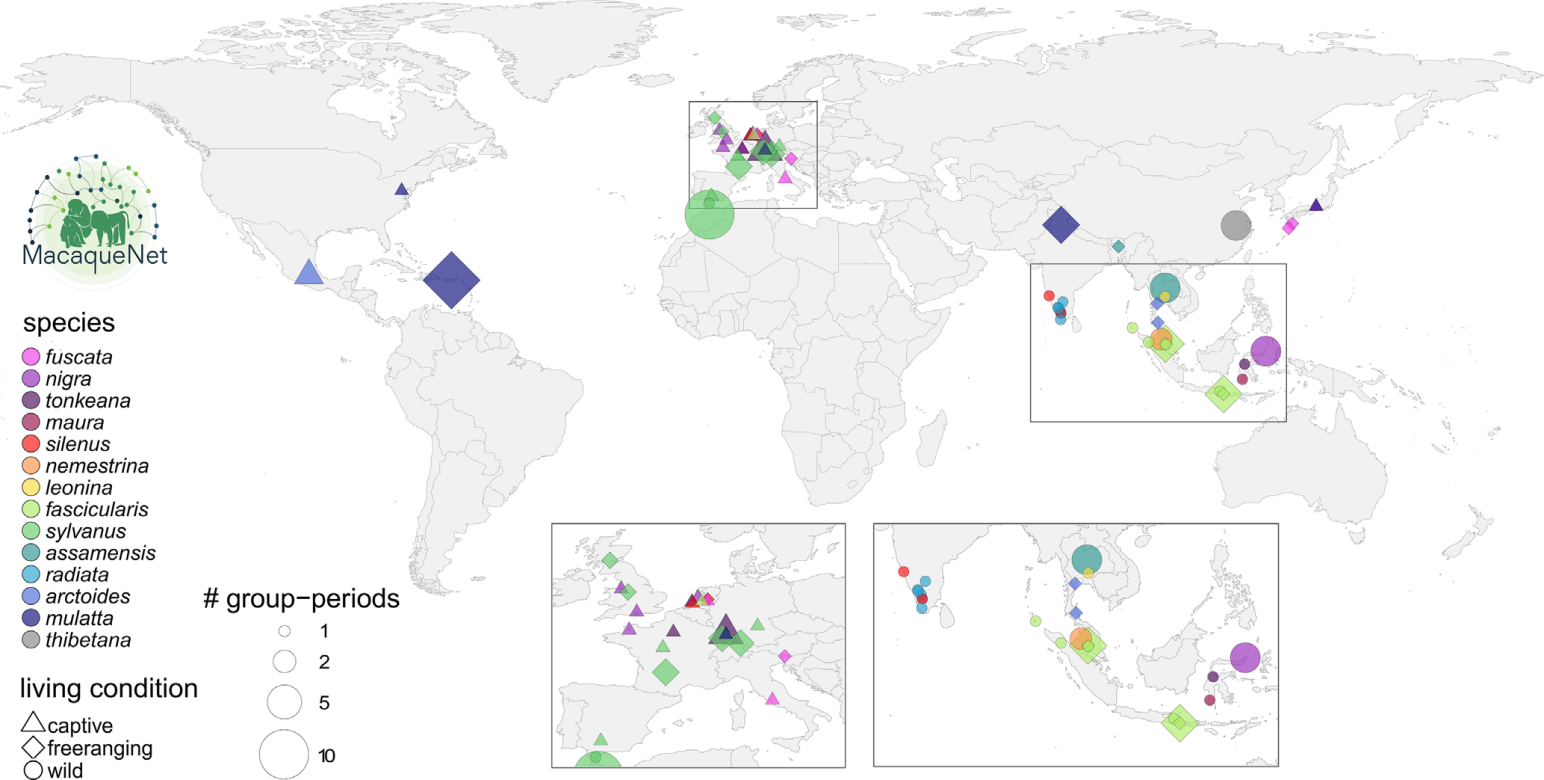
Geographical distribution of research sites in which the data currently present in the MacaqueNet database have been collected. Populations in America and Europe (with the exception of Gibraltar) have been introduced. Living conditions are classified as wild, free‐ranging or captive (see glossary at https://macaquenet.github.io/documentation/ for definitions). Group‐periods represent the total number of study periods for all groups at a given research site.

We brought together social behavioural data from independent research teams into a standardised cross‐species database. The MacaqueNet database currently contains social interaction data from 1981 to the present for 14 of the 25 recognised species of macaques (Figure 1). At the time of writing, the data have been collected on 21 wild, 22 captive and 18 free‐ranging populations (some of which have been observed for several study periods) at a total of 61 field sites, zoos and research centres (Figure 2), resulting in a total of 3972 individual behavioural datapoints (Figure 1). The full extent of the data available can be explored using the search tool on the MacaqueNet website: https://macaquenet.github.io/database/.

**FIGURE 3:**
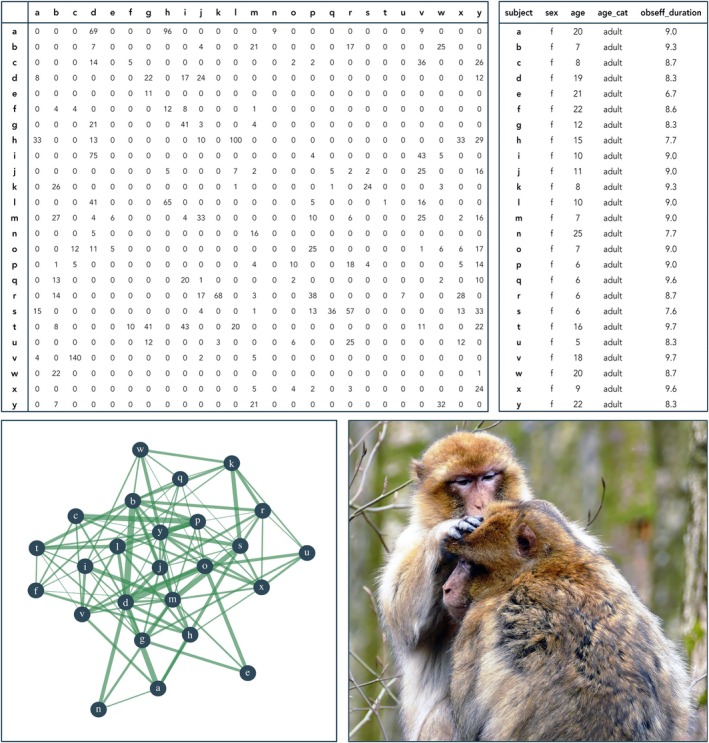
Example of the data of one dataset as stored in the MacaqueNet database. Top left: A sociometric matrix documenting pairwise interactions in a given group‐period. For directed matrices, the actors—the individuals who initiate the behaviour—are listed in the rows, while the receivers—the individuals towards whom the behaviour is directed—are listed in the columns. The matrix entries represent either the total number of times (counts, as depicted here) or the total duration (in seconds) for which an individual in the row performed the behaviour towards the individual in the column for a given study period. Depicted here: Counts of dyadic grooming events in a group of Barbary macaques (*Macaca sylvanus*) from La Montagne des Singes in 2017. Top right: A subject table listing all individuals observed in a given group‐period, along with individual attributes: Sex, age and observation effort, here duration of observation (in hours). Bottom left: An illustration of the network representing the data in the sociometric matrix. Each blue circle represents a subject, green lines between circles represent dyadic affiliation strength, here quantified as the dyadic rate of grooming. Bottom right: A picture of Barbary macaques grooming (photo credit: Anthony Poynton).

The core of the database consists of sociometric matrices that represent the two primary axes of animal social structure: dyadic affiliative and dyadic agonistic behaviour between individuals, aggregated by study period (Figure 3). The behavioural data comprise three categories of affiliative behaviour—grooming, spending time in body contact and spending time in close spatial proximity—and two categories of agonistic behaviour—contact and non‐contact aggression. These data represent the most common behavioural interactions expressed in macaques (Thierry, 2007), forming the backbone of affiliative and dominance relationships, and are therefore collected by most researchers studying macaques. Data have been collected observing one focal individual or (part of a) group at a time, using continuous recording or discrete sampling of dyadic behaviours, recorded as counts or durations (see glossary at https://macaquenet.github.io/documentation/ for definitions). Each set of sociometric matrices for a particular group‐period (i.e. a given study period for a given group) is accompanied by a subject data file, which contains individual attributes on sex, age and observation effort (Figure 3).

**FIGURE 4:**
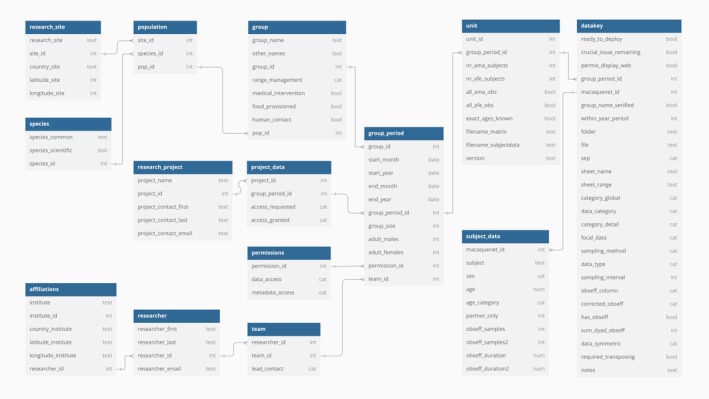
Set‐up of the relational MacaqueNet database. All variables are defined in the glossary (available at https://macaquenet.github.io/documentation/). Arrows indicate how each table is linked to at least one other table in the database through unique identifiers (the ‘‐id’ columns in each table). Each entry in the ‘datakey’ table links to a sociometric matrix with all instances of a specific behaviour category for one group period (as depicted in Figure 3 top left). Part of the ‘datakey’ table is only relevant for the data import pipeline. This figure was made using dbdiagram (https://www.dbdiagram.io/).

In addition to the behavioural data, the database includes rich metadata on methodology, study populations and research teams. The data and metadata are organised as a relational database (Figure 4), with clear definitions for each variable outlined in a glossary (both the relational database structure and the glossary are available at https://macaquenet.github.io/documentation/).

**FIGURE 5:**
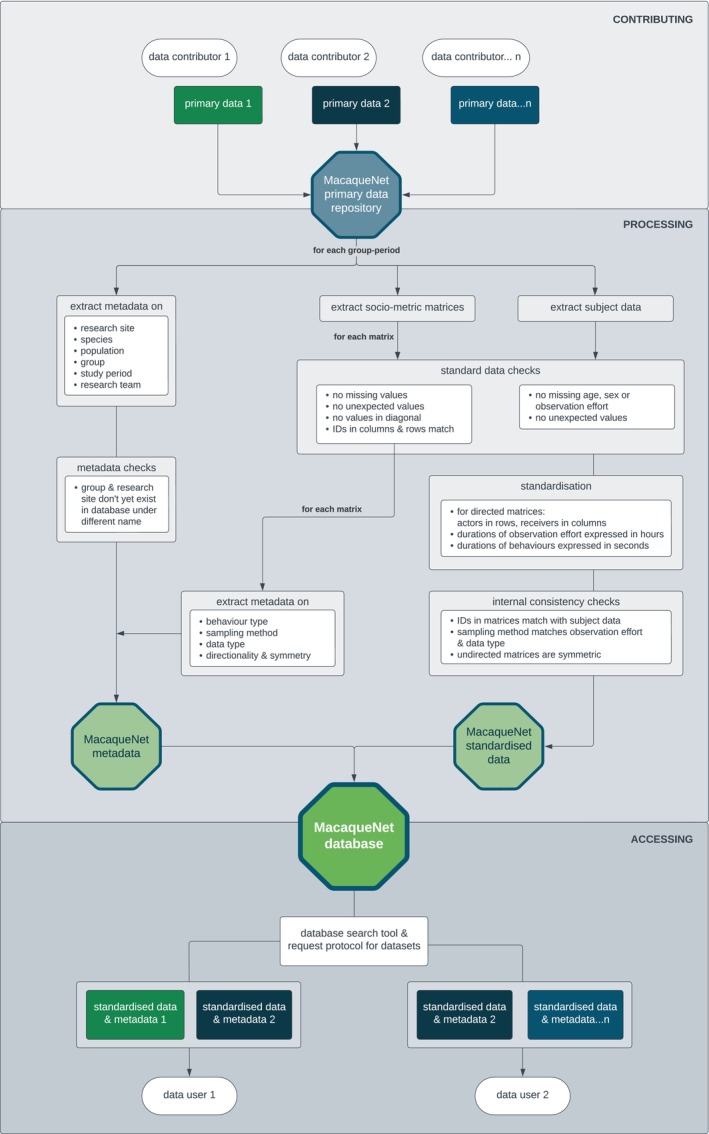
Overview of the workflow from primary data submission, over the data checks and standardisation pipeline to data requests. All contributed data contain several sociometric matrices along with corresponding subject data and metadata for at least one group‐period for a specific species.

To ensure that the data and metadata are comparable across studies, we set up a standardisation pipeline through which all contributed data (‘primary data’) is run before it enters the database. Figure 5 provides an overview of the workflow from primary data submission, over the data checks and standardisation pipeline to data requests.

In a nutshell, for each dataset contributed to MacaqueNet, we extract the sociometric matrices for each behaviour, the subject data and the metadata on the study population, observational methods and research team. We perform standard data checks on the extracted sociometric matrices and subject data to ensure there are no missing or unexpected values. Next, we standardise the data by establishing uniform units of measurement and clearly defined categories for metadata on the populations and observation protocols. For instance, we classify observational methods based on three descriptors (see glossary at https://macaquenet.github.io/documentation/ for full definitions): ‘focal_data’ (whether the data are collected focusing on one individual or a group of individuals), ‘sampling_method’ (whether the data are recorded continuously or at set time intervals) and ‘data_type’ (whether the data are collected as counts or as durations). Similarly, we classify groups based on four descriptors (see glossary for full definitions): ‘range_management’ (the extent to which a group's range is managed), ‘medical_intervention’ (whether a group receives medical interventions as part of the population's management plan), ‘food_provisioned’ (whether a group is routinely provided with food) and ‘human_contact’ (whether a group is in regular contact with non‐researcher humans). This classification process is essential because terms describing observation methods and population settings often have multiple inconsistent definitions (e.g. ‘focal sampling’ or ‘free‐ranging’). Our collaborative approach to building the database was particularly powerful in this regard, as it allowed us to standardise data through direct communication with contributors, thus avoiding misinterpretations. The resulting standardisation ensures consistency across datasets, simplifies filtering for data users based on their specific needs and facilitates accurate comparison and analysis.

Both the primary data and the standardised database are currently stored and managed in GitHub. The data standardisation pipeline is available as an R package https://github.com/MacaqueNet/macaquenetpipeline), which provides examples and code for how contributed data are processed before entering the database. The available data can be explored and requested using the search tool on the MacaqueNet website (https://macaquenet.github.io/database/), which also provides a version history documenting changes and updates to the database.

### 2.4 MacaqueNet is a community that facilitates comparative research

MacaqueNet makes macaque data FAIR (Wilkinson et al., 2016), in four main ways. First, by identifying, accessing and bringing together data from multiple sources into one centralised place https://macaquenet.github.io/database/. Instead of creating a database of summarised traits with one data point per species, this approach consolidates the original data itself and creates a more flexible resource for future use. Moreover, with several species represented by multiple populations and/or study periods, both between‐species and within‐species variation are captured (Mocha et al., 2024; Sinha, 2005). The latter is often overlooked in comparative analyses but can be a significant source of error if not accounted for (Sandel et al., 2016; Schradin, 2013). Second, by developing a common vocabulary for the data and metadata in the MacaqueNet database, with openly accessible definitions (Borries et al., 2016; Urbano & Cagnacci, 2021). Third, by standardising data across studies and research sites together with the data contributors who understand the intricacies and subtleties of the data (Schneider et al., 2019; Urbano & Cagnacci, 2021). Fourth, by providing transparent and replicable annotation of how primary data were processed so that the original data can always be recovered and processed in different ways if future researchers want to make other decisions (Schneider et al., 2019; Urbano & Cagnacci, 2021). As a result, MacaqueNet provides a meticulously curated database with data readily available for future comparative studies (Borries et al., 2016; Gomes et al., 2022).

MacaqueNet extends beyond mere data sharing. By linking researchers across different research groups and institutions, MacaqueNet facilitates a global exchange of ideas and fosters the development of new research projects. To provide an opportunity for all the current data contributors to meet, discuss their research and learn about ongoing projects, we organised a virtual global symposium ‘Weaving the MacaqueNet’ in November 2021. During the symposium, future directions for MacaqueNet were discussed and agreed on. As a first step in the establishment of an infrastructure that permits researchers to communicate and coordinate their research, we set up a website (https://macaquenet.github.io), a mailing list and a group messaging channel on Slack. The second ‘Weaving the MacaqueNet’ symposium was held in August 2023 at the Joint Conference of the International Primatological Society and Malaysian Primatological Society, where the MacaqueNet community got the opportunity to meet in person. At that meeting, we discussed the expansion of MacaqueNet to include more types of data, thereby increasing MacaqueNet's ability to address a range of questions on social evolution. As a result, efforts are currently underway to include ecological, life history and genetic data to complement the existing behavioural data. To promote transparency, we also agreed to make all meetings associated with MacaqueNet open to all MacaqueNet members, in addition to disseminating a quarterly newsletter outlining new projects and discussion points.

New projects can be easily proposed, and data requested with a brief project proposal via the website (https://macaquenet.github.io/database/). The proposal is forwarded to the data contributors, who assess whether they can provide their data for the proposed project and determine their usage terms (see MacaqueNet terms of use at https://macaquenet.github.io/documentation/). New types of data to be added to the database can also be suggested on the website. These ideas and their potential implementation are discussed at each MacaqueNet symposium.

The code for the website, the glossary, the relational database structure, the workflow and the terms of use are openly accessible in a GitHub repository (https://github.com/MacaqueNet). Together, these resources provide a fully replicable model for the development of cross‐species collaborative databases.

## 3 DISCUSSION

In this paper, we advocate for the collaborative building of FAIR cross‐species databases as a novel and promising tool in comparative research. Understanding the ecological and evolutionary drivers of variation in social behaviour requires comparing taxa with distinct life histories, ecology and interlinked evolutionary histories (Kappeler et al., 2019; Lukas & Clutton‐Brock, 2018; Pinter‐Wollman et al., 2013). Yet, despite decades worth of social behavioural data (Sheldon et al., 2022), substantial challenges have impeded systematic comparative research (Albery et al., 2024; De Moor et al., 2024), one of which is the creation of truly comparable databases (Borries et al., 2016). We propose that large‐scale collaborative efforts, where data and expertise are shared among researchers, can provide a solution to this issue and help facilitate reliable cross‐species comparisons. To inspire and facilitate more collaborative efforts in behavioural research, we share our experience in developing MacaqueNet and its cross‐species database.

Building on the foundations of previous big‐team projects that have been instrumental in shaping MacaqueNet (including but not restricted to SPI‐Birds: Culina et al., 2020; ManyPrimates: ManyPrimates et al., 2019), we highlight three substantial advantages for the scientific community to join forces and pool their data into FAIR databases.

First, by creating an enduring infrastructure for collaboration, big‐team science efforts represent a crucial interface linking existing data and data users (Culina et al., 2020; Urbano & Cagnacci, 2021). They ensure that valuable data, which might have otherwise remained largely unused, are easier to discover, repurpose, and synthesise (Gonzalez & Peres‐Neto, 2015; Purgar et al., 2022; Wallis et al., 2013) and help pinpoint areas that would benefit most from additional data collection. Moreover, rather than summarising the data into single datapoints, building a database that brings the standardised original data together creates a much more flexible resource. Such a database represents variation at different biological levels, can easily keep growing to include more data as they become available, and can be subset and summarised tailored to specific research questions.

Second, much of the existing social behaviour data are the result of years of effort and funding acquisition to set up the necessary logistics and to collect data on often wild and endangered animals. Big‐team science can set up guidelines and regulations to ensure that sharing is done in such a way that it considers individual researchers' interests, and their ability to acquire funding to keep collecting new data. Transparent but controlled accessibility, where data contributors retain full ownership of their data, best balances benefits to the scientific community while protecting data contributors, as proven successful in other big‐team science endeavours (Culina et al., 2020; Strier et al., 2010; Urbano & Cagnacci, 2021). Creating a community rather than just a database also offers data contributors networking opportunities and access to others' data, fostering collaborative research possibilities (Urbano & Cagnacci, 2021).

Third, social behavioural data are particularly complex, with many researcher degrees of freedom involved in how the data are defined and what methods of observation are used (Borries et al., 2016). Taking a collaborative approach to building comparative databases allows for data standardisation to be done through direct interaction with data contributors, thus ensuring clarity around the intricacies of the data and averting inadvertent misinterpretations (Mocha et al., 2024; Schradin, 2017; Urbano & Cagnacci, 2021). This process is essential for providing database users with the necessary information to determine which data are comparable for their specific research questions and how to appropriately account for differences when conducting comparisons (De Moor et al., 2024).

The rise of collaborative databases in a variety of fields is a testament to the enthusiasm of researchers to join forces and share data and resources to facilitate comparative and interdisciplinary science, an emerging and promising way of doing research (Coles et al., 2022). Large‐scale collaborative endeavours like MacaqueNet allow for the exploration of innovative and comparative questions no single research group could address individually, make testing the reproducibility of research findings across contexts possible, and make data access more equitable by setting up FAIR (Wilkinson et al., 2016) and transparent data sharing policies. Sustaining such initiatives necessitates continual financial and personnel support for database storage and maintenance as well as community engagement. This underscores the importance for funding agencies and the broader academic system to prioritise and fund these endeavours accordingly (Gomes et al., 2022). While the creation of databases on behavioural data comes with substantial challenges, we truly believe this is the way forward to harness the incredible comparative potential of the wealth of existing behavioural data. We hope that MacaqueNet, along with similar endeavours, can catalyse large‐scale collaborative research on animal behaviour.

## AUTHOR CONTRIBUTIONS

Julie Duboscq, Christof Neumann, Lauren J. N. Brent, Julia Ostner and Oliver Schülke generated the idea to compile data for comparative research. Julie Duboscq and Christof Neumann instigated the collaborative effort, with Lauren J. N. Brent and Delphine De Moor formalising it into the grassroots network ‘MacaqueNet’; Delphine De Moor, Lauren J. N. Brent, Julie Duboscq and Macaela Skelton built a community of researchers. Delphine De Moor, Julie Duboscq and Macaela Skelton gathered the data. Christof Neumann and Delphine De Moor created the database package and standardised the data. Delphine De Moor wrote the manuscript, with input from Lauren J. N. Brent, Julie Duboscq, Christof Neumann, Julia Ostner, Oliver Schülke and Krishna N. Balasubramaniam. Delphine De Moor, Macaela Skelton and Christof Neumann created the figures. All authors contributed data to the MacaqueNet database, provided critical feedback on the manuscript's structure and content, and gave their final approval for publication.

## CONFLICT OF INTEREST STATEMENT

The authors declare that they have no competing interests.

## STATEMENT ON INCLUSION

One of MacaqueNet's primary objectives is to foster collaboration among macaque researchers worldwide. The geographic distribution of MacaqueNet and the authors involved in this manuscript already reflects a relatively diverse group of researchers. However, we remain committed to enhancing the inclusion of researchers from macaque‐range countries in Asia and Northern Africa. We aspire to see the MacaqueNet community play a pivotal role in achieving this goal by establishing a network where funding opportunities and access to data are more equitably distributed. Furthermore, we aim to provide a platform within MacaqueNet to showcase the remarkable work conducted by researchers in macaque‐range countries and to catalyse collaborations on a global scale.

## Data Availability

The code for the data standardisation pipeline on the available datasets, along with resources, such as the Glossary, the workflow and the MacaqueNet Terms of Use, are openly accessible in the MacaqueNet GitHub repository (https://github.com/MacaqueNet/). MacaqueNet data can be requested following the requesting protocol through the MacaqueNet website (https://macaquenet.github.io/database/).
